# Hypoalbuminaemia and One-Year Mortality in Haemodialysis Patients with Heart Failure: A Cohort Analysis

**DOI:** 10.3390/jcm10194518

**Published:** 2021-09-29

**Authors:** Ana Cardoso, Carolina Branco, Mariana Sant’Ana, Cláudia Costa, Bernardo Silva, José Fonseca, Cristina Outerelo, Joana Gameiro

**Affiliations:** 1 Centro Hospitalar Universitário Lisboa Norte, Department of Medicine, Division of Internal Medicine II, EPE, Av. Prof. Egas Moniz, 1649-035 Lisboa, Portugal; anaacardoso4@gmail.com; 2 Centro Hospitalar Universitário Lisboa Norte, Department of Medicine, Division of Nephrology and Renal Transplantation, EPE, Av. Prof. Egas Moniz, 1649-035 Lisboa, Portugal; carolinagbranco@hotmail.com (C.B.); claucosta_30@hotmail.com (C.C.); bmsilva@campus.ul.pt (B.S.); jose.nuno.agapito@gmail.com (J.F.); cristinaouterelo@gmail.com (C.O.); 3 Clínica Universitária de Nefrologia, Faculdade de Medicina da Universidade de Lisboa, Universidade de Lisboa, Av. Prof. Egas Moniz, 1649-035 Lisboa, Portugal; marianamatosana@campus.ul.pt

**Keywords:** chronic haemodialysis, heart failure, mortality, malnutrition, hypoalbuminaemia

## Abstract

Introduction: The prevalence of chronic kidney disease (CKD) and heart failure (HF) has been rising over the past decade, with a prevalence close to 40%. Cardiovascular disease and malnutrition are common comorbidities and known risk factors for mortality in haemodialysis (HD) patients. We aimed to evaluate the one-year mortality rate after dialysis induction, and the impact of serum albumin levels on survival outcomes, in patients with CKD and HF. Methods: This was a retrospective analysis of patients with CKD and HF who underwent chronic HD between January 2016 and December 2019 in a tertiary-care Portuguese hospital. Variables were submitted to univariate and multivariate analysis to determine factors predictive of one-mortality after HD start. Results: In total, 204 patients were analysed (mean age 75.1 ± 10.3 years). Within the first year of HD start, 28.7% of patients died. These patients were significantly older [79.8 ± 7.2 versus 72.9 ± 10.9 years, *p* < 0.001; OR 1.08 (1.04–1.13), *p* < 0.001] and had a higher mean Charlson Index [9.0 ± 1.8 versus 8.3 ± 2.0, *p* = 0.015; OR 1.22 (1.04–1.44), *p* = 0.017], lower serum creatinine [5.1 ± 1.6 mg/dL versus 5.8 ± 2.0 mg/dL; *p* = 0.021; OR 0.80 (0.65–0.97), *p* = 0.022], lower albumin levels [3.1 ± 0.6 g/dL versus 3.4 ± 0.6 g/dL, *p* < 0.001; OR 0.38 (0.22–0.66), *p* = 0.001] and started haemodialysis with a central venous catheter more frequently [80.4% versus 66.2%, *p* = 0.050]. Multivariate analysis identified older age [aOR 1.07 (1.03–1.12), *p* = 0.002], lower serum creatinine [aOR 0.80 (0.64–0.99), *p* = 0.049] and lower serum albumin [aOR 0.41 (0.22–0.75), *p* = 0.004] as predictors of one-year mortality. Conclusion: In our cohort, older age, lower serum creatinine and lower serum albumin were independent risk factors for one-year mortality, highlighting the prognostic importance of malnutrition in patients starting chronic HD.

## 1. Introduction

The global prevalence of chronic haemodialysis (HD) is 298.4/pmp [1]. Chronic kidney disease (CKD), mainly end-stage renal disease (ESRD), is recognised as a serious risk factor for mortality. HD is the major treatment modality for renal replacement therapy worldwide and has the highest mortality rate, with 40% of patients surviving for five years in a large prospective United States-based study. Overall, mortality is 10–20 times higher than in the general population, and annual mortality is around 9% per year [2,3].

Cardiovascular disease (CVD) is the main cause of death in patients with ESRD on HD, present in >50% of patients undergoing dialysis, and the relative risk of death due to CVD events in HD is reported to be 20 times higher than in the general population. This is likely due to ventricular hypertrophy, as well as non-traditional risk factors, such as chronic volume overload, anaemia, inflammation, oxidative stress and chronic kidney disease–mineral bone disorder [4].

The prevalence of CKD and heart failure (HF) has been rising over the past decade, with higher rates in patients who have ESRD, and a prevalence of close to 40%, given the common soil of traditional cardiovascular disease risk factors such as diabetes, hypertension and obesity, as well as the non-traditional risk factors as mentioned above [5]. In a historic United States-based study of more than 1900 patients in HD, the incidence of HF was 71 per 1000 person-years, with an 83% mortality rate at 3 years. The additional diagnosis of HF in ESRD patients adds complexity to the volume status assessment and optimal fluid management techniques [6].

Protein–energy malnutrition is a major risk factor for mortality and inflammation, with serum albumin being the main marker of nutrition and inflammation. Studies have suggested that a serum albumin level of less than 3.8 g/dL (and/or a reduction in serum albumin levels) leads to a greater mortality risk in patients with ESRD, as well as in other diseases [7]. Aside from being a powerful prognostic marker due to malnutrition and inflammation, evidence is growing that hypoalbuminaemia is linked to the emergence of several CVDs, such as HF, with a prevalence of hypoalbuminaemia ranging from 20 to 25% in chronic HF to 90% in elderly patients with acute HF [8].

In our study, we aimed to evaluate the one-year mortality rate after dialysis induction and the impact of serum albumin levels on outcomes in patients with chronic kidney disease (CKD) and heart failure (HF).

## 2. Materials and Methods

This study is a retrospective analysis of patients who initiated HD between January 2016 and December 2019 in Centro Hospitalar Universitário Lisboa Norte (CHULN) in Lisbon, Portugal. The Ethical Committee approved this study, in agreement with institutional guidelines. Informed consent was waived, given the retrospective and non-interventional nature of the study.

### 2.1. Participants

We selected, under our eligibility criteria, all adult patients (≥18 years of age) with chronic kidney disease (CKD) and concomitant heart failure (HF) who initiated haemodialysis from 1 January of 2016 to 31 December of 2019. We considered both urgent and planned renal replacement therapy (RRT) start. Patients with previous RRT, namely peritoneal dialysis or renal transplant, were excluded, as were patients lost to follow-up.

### 2.2. Variables and Outcomes

Data were obtained from individual electronic clinical records. The following variables were collected: demographic characteristics (age, gender); comorbidities [CKD, HF, diabetes mellitus, hypertension, ischaemic cardiomyopathy, cerebrovascular disease, rheumatic disease, chronic hepatic disease, chronic obstructive pulmonary disease (COPD), active malignancy, dementia]; HD access (central venous catheter, arteriovenous fistula, arteriovenous graft); laboratory at HD start [haemoglobin, neutrophil and lymphocyte count, platelet count, serum albumin, serum ferritin, serum parathyroid hormone (PTH), SCr, estimated glomerular filtration rate (eGFR), serum urea, C-reactive protein (CRP)].

The primary outcome was mortality within one year of HD induction.

### 2.3. Definitions

The estimated glomerular filtration rate (eGFR) was calculated using the Chronic Kidney Disease Epidemiology Collaboration (CKD-EPI) creatinine equation. Presence of CKD was defined as an eGFR lower than 60 mL/min/1.73 m^2^.

Heart failure was considered based on previously known clinical diagnosis of any cause. Diabetes mellitus was defined in accordance with the American Diabetes Association Guidelines. Arterial hypertension was diagnosed according to the European Society of Cardiology and European Society of Hypertension Guidelines. Ischaemic cardiomyopathy included both previous myocardial infarction and chronic coronary artery disease, and was based on prior diagnosis. COPD included emphysema and chronic bronchitis. Cerebrovascular disease was defined based on prior history of stroke, carotid, vertebral or intracranial stenosis, aneurysms or vascular malformations. Peripheral arterial disease and dementia were considered based on previously documented clinical diagnosis. Rheumatic disease included all previously diagnosed autoimmune and inflammatory diseases. Chronic liver disease was defined as deterioration of liver function for more than six months from all causes, as previously documented in the clinical history.

### 2.4. Statistical Methods

Categorical variables were described as the total number and percentage of each category, while continuous variables were described as the mean ± standard deviation. The Kolmogorov–Smirnov normality test was used to examine if variables were normally distributed. Continuous variables were compared using Student’s *t*-test, whereas categorical variables were compared using Chi-square test.

All variables were submitted to univariate analysis to find statistically significant factors that could be predictive of mortality within the first year of HD induction. Subsequently, variables with a significant statistical difference underwent multivariate analysis using the Cox logistic regression method. Data were conveyed as odds ratios (OR) with 95% confidence intervals (CI). Statistical significance was established as a *p*-value of less than 0.05. Statistical analysis was achieved using the statistical software package SPSS for Windows (version 21.0).

## 3. Results

A total of 204 CKD patients with concomitant heart failure began HD during the study period. The baseline characteristics are described in Table 1.

The mean age of the population was 75.1 ± 10.3 and a majority of patients were male (60.3%) and Caucasian (92.6%). Overall, there was a large prevalence of hypertension (88.7%), diabetes mellitus (58.8%) and ischaemic cardiomyopathy (44.6%). In total, 49 patients had peripheral artery disease, 42 patients had cerebrovascular disease, 11 had dementia and 35 patients had neoplasia. The mean Charlson Index was 8.5 ± 2.0. In total, 65% of patients (*n* = 133) had already had previous regular nephrology follow-ups before initiating haemodialysis.

At dialysis initiation, mean haemoglobin was 9.7 ± 1.6 g/dL, with almost 55% of patients with haemoglobin lower than 10 g/dL. Mean serum urea was 192.2 ± 61.5 mg/dL, mean serum creatinine 5.6 ± 1.9 mg/dL and mean eGFR 9.5 ± 4.1 mL/min/1.73 m^2^. Mean albumin was 3.3 ± 0.6 g/dL, with more than half of patients with hypoalbuminaemia. Mean PTH was 254.0 ± 109.2 pg/mL, mean ferritin 482.8 ± 252.2 μg/L and mean CRP 5.1 ± 2.5 mg/dL.

Regarding vascular access at dialysis initiation, 70.6% was induced through a central venous catheter, 27.5% through an arteriovenous fistula and 2.0% through an arteriovenous graft.

The mean follow up was 23.1 ± 16.0 months.

During this follow-up, 105 patients (51.5%) died. Fifty-six patients (28.7%) died within the first year of starting HD.

### 3.1. Hypoalbuminaemia

In total, 107 patients had serum albumin lower than 3.5 g/dL, the majority of whom were male (57.0%) and Caucasian (96.7%), as seen in Table 2. Their mean age was 76.2 ± 11.4 years.

Patients with hypoalbuminaemia had significantly lower serum haemoglobin (9.3 ± 1.7 versus 10.1 ± 1.5; *p* = 0.001) and eGFR (8.7 ± 3.4 versus 10.4 ± 4.6; *p* = 0.003), and higher serum ferritin (603.0 ± 221.0 versus 333.7 ± 185.7; *p* = 0.009) and CRP (7.3 ± 2.4 versus 2.5 ± 1.7; *p* < 0.001). They also fulfilled sepsis criteria more often than patients without hypoalbuminaemia (57.9% versus 25.5%; *p* < 0.001).

One-year mortality after starting HD was higher in patients with hypoalbuminaemia (35.5% versus 20.5%; *p* = 0.021).

### 3.2. Early Mortality

Patients who died within the first year of starting HD were significantly older (79.8 ± 7.2 versus 72.9 ± 10.9 years, *p* < 0.001; unadjusted odds ratio (OR) 1.08 (1.04–1.13), *p* < 0.001), without differences in gender or race.

Although the mortality group had a higher mean Charlson Index (9.0 ± 1.8 versus 8.3 ± 2.0, *p* = 0.015; OR 1.22 (1.04–1.44), *p* = 0.017), there was no statistically significant difference regarding the prevalence of each comorbidity.

Regarding laboratory values at haemodialysis initiation, one-year mortality was higher in patients with lower serum creatinine (5.1 ± 1.6 mg/dL versus 5.8 ± 2.0 mg/dL; *p* = 0.021; OR 0.80 (0.65–0.97), *p* = 0.022) and albumin levels (3.1 ± 0.6 g/dL versus 3.4 ± 0.6 g/dL; *p* < 0.001; OR 0.38 (0.22–0.66, *p* = 0.001). One-year mortality was also higher in patients who started haemodialysis with a central venous catheter (*n* = 45, 80.4% versus *n* = 92, 66.2%; *p* = 0.050).

In a multivariate analysis, older age [adjusted odds ratio (aOR) 1.07 (1.03–1.12), *p* = 0.002], lower serum creatinine [aOR 0.80 (0.64–0.99), *p* = 0.049] and lower serum albumin [aOR 0.41 (0.22–0.75), *p* = 0.004] were independent predictors of early mortality (Table 3).

## 4. Discussion

In our cohort of HF patients who initiated chronic HD, one-year mortality was highest in older patients (79.8 ± 7.2 versus 72.9 ± 10.9 years, *p* < 0.001; aOR 1.07 (1.03–1.12), *p* = 0.002), as well as in patients with lower serum albumin levels (3.1 ± 0.6 g/dL versus 3.4 ± 0.6 g/dL; *p* < 0.001; aOR 0.38 (0.22–0.66, *p* = 0.001) and lower serum creatinine levels (5.1 ± 1.6 mg/dL versus 5.8 ± 2.0 mg/dL; *p* = 0.021; aOR 0.80 (0.65–0.97), *p* = 0.022).

Mortality in HD patients remains high and has been reported in up to 20% of patients per year in the United States. A study including 18,707 incident HD patients demonstrated that up to 36% of patients died within the first 24 months. Early death was associated with more advanced age, higher proportion of CVD and higher prevalence of cardiovascular diseases [9].

Indeed, more than half of reported deaths are due to cardiovascular disease, and HF is a common cardiovascular condition at HD initiation. Having both CKD and HF increases the risk of hospitalisation, need for intensive care or RRT and mortality [10]. Moreover, these patients often fail to respond to conventional therapies, experience increased toxicity and are less likely to be treated due to concerns about hypotension, kidney function and hyperkalaemia [10].

Stack et al. conducted a retrospective study of 926,298 incident HD patients and demonstrated that one third of patients had HF, that the prevalence of HF was higher in the female gender and that the prevalence increased with advancing age [11]. Mortality was significantly higher in HF patients (74.4% versus 54.2%). Interestingly, despite remaining significantly high, the mortality rate of these patients declined from 1995 to 2005, potentially owing to temporal improvements in overall kidney disease and cardiovascular care [11].

Another study by Raja et al. also demonstrated that left-sided HF significantly contributed to mortality in ESRD patients over a 2.8-year follow-up (52 versus 32%, HR 1.95 (95%CI 1.25–3.06)) [12]. In a retrospective study, Molnar et al. identified a mortality rate as high as 67.8% over a median follow-up of 2.4 years in HF patients who underwent HD [13]. In our study, 51.5% died during the overall follow-up and 28.7% died within the first year of starting HD.

Considering the increased risk of mortality of HD patients with HF, it is crucial to identify risk factors in these patients. In HD patients, white skin, older age, low serum albumin levels, low and elevated serum phosphorus levels and anaemia have all been identified as risk factors for mortality over the first year of HD initiation [14]. Hypoalbuminaemia has emerged as a prognostic parameter in patients with many cardiovascular diseases and in HD patients [8,15].

The decrease in albumin may be a powerful prognostic marker, mainly as a result of malnutrition and inflammation [7]. One of the most serious clinical issues encountered in ESRD patients is the co-occurrence of malnutrition, inflammation and atherosclerosis (MIA syndrome). MIA syndrome is multifactorial, with possible causes including reduced nutrient intake and prescribed dietary restrictions, catabolic comorbid illnesses, anorexia, acidaemia, endocrine disorders, nutrient loss through dialysis, uremic toxins, decreased clearance of inflammatory cytokines, increased production of inflammatory cytokines and increased oxidative stress, volume overload, dialysis adequacy, bio-incompatibility and purity of the dialysate, anaemia and hyperparathyroidism [16]. The prevention of MIA syndrome is of utmost importance due to its important association with cardiovascular disease, worsening survival potential [17,18,19].

The U.S. Dialysis Outcomes and Practice Patterns Study (DOPPS) showed that mortality risk increases correspondingly to a decline in the serum albumin when it falls below 4.0 g/dL [20,21]. In the HEMO Study, an increase in serum albumin reduced the relative risk of mortality for more than 6 months of follow-up in patients with albumin <3.5 g/dL [15]. In this study, higher serum CRP concentrations were also associated with increased risk of mortality [15]. A 10-year cohort study from Japan reported that patients with serum albumin levels >3.8 g/dL consistently had better survival rates [22]. Lukowsky also demonstrated that hypoalbuminaemia <3.5 mg/dL was associated with 33% of all deaths within the first 90 days of HD initiation [9].

In our study, we report that hypoalbuminaemia is an independent risk factor for early mortality in HD patients with HF (35.5% versus 20.5%; *p* = 0.021). Additionally, patients with hypoalbuminaemia also had increased levels of inflammatory markers (higher serum ferritin (*p* = 0.009) and CRP (*p* < 0.001)), which demonstrated the association between malnutrition and inflammation with mortality and explained why inflammatory markers were not independently associated with mortality in our cohort.

Lower levels of serum creatinine also reflect low protein intake and/or decreased muscle mass, and may indicate a need for assessment of protein–energy status [23]. In a retrospective study of HD patients, the authors found that serum creatinine concentration correlated well with dual-energy X-ray absorptiometry (DEXA)-measured lean body mass, concluding that serum creatinine can serve as a reliable muscle mass biomarker [24]. Another study that focused on the association between nutritional status and clinical outcomes in dialysis patients found that, during the 15-year study period, patients who survived had higher enrolment levels of nutritional markers, namely serum creatinine [25].

Our study also demonstrated a significant association between lower creatinine levels and increased mortality risk.

There are some important virtues to be noted in our study. To our knowledge, this is the first study to demonstrate the impact of lower serum creatinine and lower serum albumin as independent risk factors for one-year mortality, thus highlighting the prognostic importance of malnutrition in patients starting HD. Moreover, despite its retrospective design, the studied variables were routinely recorded in daily practice, which allowed for the analysis of important covariates with impact on outcomes. Serum albumin concentration is a simple and inexpensive routine laboratory test, which provides relevant prognostic information in cardiorenal patients.

We must take into account several limitations of our study. First, this is a single-centre, retrospective study which limits generalisation. The small size of our cohort may have compromised, at least in part, our conclusions. We did not assess ejection fraction and analyse mortality accordingly, which could influence results. Finally, we could not determine the exact mechanisms contributing to mortality in these patients, and causes of mortality were not ascertained.

To conclude, in this cohort of HF patients who initiated chronic HD, one-year mortality was higher in older patients and in patients with lower serum albumin levels and lower serum creatinine levels, highlighting the prognostic importance of malnutrition in these patients. A routine assessment on patients’ nutritional status is important to estimate the prognosis at HD initiation. Further prospective studies are required to address the role of hypoalbuminaemia as a predictor of mortality in patients in chronic HD with HF.

## Figures and Tables

**Table 1:** Baseline characteristics.

Characteristic	Total (*n* = 204)	One Year Mortality (*n* = 56)	Survival (*n* = 139)	*p* Value
Age (year)	75.1 ± 10.3	79.8 ± 7.2	72.9 ± 10.9	<0.001
Gender (Male)—*n* (%)	123 (60.3)	32 (57.1)	84 (60.4)	0.672
Race (Caucasian)—*n* (%)	189 (92.6)	54 (96.4)	127 (91.4)	0.215
Comorbidities—*n* (%)				
Hypertension	181 (88.7)	47 (83.9)	126 (90.6)	0.180
Diabetes	120 (58.8)	31 (55.4)	86 (61.9)	0.401
Ischemic cardiomyopathy	91 (44.6)	28 (50.0)	63 (45.3)	0.554
COPD	37 (18.1)	12 (21.4)	24 (17.3)	0.498
Cerebrovascular disease	42 (20.6)	12 (21.4)	28 (20.1)	0.841
Peripheral artery disease	49 (24.0)	15 (26.8)	34 (24.5)	0.735
Dementia	11 (5.4)	3 (5.4)	8 (5.8)	0.913
Neoplasia	35 (17.2)	11 (19.6)	21 (15.1)	0.439
Chronic liver disease	8 (3.9)	2 (3.6)	6 (4.3)	0.812
Rheumatologic disease	11 (5.4)	4 (7.1)	6 (4.3)	0.418
Charlson Index	8.5 ± 2.0	9.0 ± 1.8	8.3 ± 2.0	0.015
Laboratory at dialysis start				
Haemoglobin (g/dL)	9.7 ± 1.6	9.7 ± 1.5	9.7 ± 1.6	0.923
Serum Urea (mg/dL)	192.2 ± 61.5	194.6 ± 68.5	191.8 ± 57.6	0.771
Serum Creatinine (mg/dL)	5.6 ± 1.9	5.1 ± 1.6	5.8 ± 2.0	0.021
eGFR (mL/min/1.73 m^2^)	9.5 ± 4.1	10.2 ± 4.6	9.2 ± 3.9	0.134
Albumin (g/dL)	3.3 ± 0.6	3.1 ± 0.6	3.4 ± 0.6	<0.001
PTH (pg/mL)	254.0 ± 109.2	251.0 ± 100.3	262.1 ± 100.6	0.737
Ferritin (ng/mL)	482.8 ± 252.2	600.0 ± 284.7	443.2 ± 273.0	0.195
CRP (mg/dL)	5.1 ± 2.5	6.5 ± 2.6	4.6 ± 2.5	0.07
Hypoalbuminemia—*n* (%)	107 (52.5)	38 (67.9)	69 (49.6)	0.021
Ferropenia—*n* (%)	69 (33.8)	19 (43.2)	50 (45.5)	0.798
Sepsis at dialysis start—*n* (%)	83 (40.7)	30 (53.6)	53 (38.1)	0.048
Vascular access at dialysis start—*n* (%)				
Central venous catheter	144 (70.6)	45 (80.4)	92 (66.2)	0.05
Arteriovenous fistula	56 (27.5)	11 (19.6)	43 (30.9)	0.111
Arteriovenous graft	4 (2.0)	0 (0.0)	4 (2.9)	0.2
Outcomes				
Follow-up (months)	23.1 ± 16.0			
One-year mortality—*n* (%)	56 (28.7)			

**Table 2:** Patient characteristics according to hypoalbuminaemia.

Characteristic	Hypoalbuminemia (*n* = 107)	Normoalbuminemia (*n* = 94)	*p* Value
Age (year)	76.2 ± 11.4	73.8 ± 8.9	0.105
Gender (Male)—*n* (%)	61 (57.0)	62 (70.0)	0.126
Race (Caucasian)—*n* (%)	103 (96.7)	86 (91.5)	0.558
Comorbidities—*n* (%)			
Hypertension	100 (93.5)	81 (86.2)	0.286
Diabetes	60 (56.1)	60 (63.8)	0.179
Ischemic cardiomyopathy	49 (45.8)	42 (44.7)	0.985
COPD	21 (19.6)	16 (17.0)	0.702
Cerebrovascular disease	26 (24.3)	16 (17.0)	0.244
Peripheral artery disease	27 (25.2)	24 (25.5)	0.871
Dementia	8 (7.5)	3 (3.2)	0.198
Chronic liver disease	6 (5.6)	2 (2.1)	0.222
Laboratory at dialysis start			
Haemoglobin (g/dL)	9.3 ± 1.6	10.1 ± 1.5	0.001
eGFR (mL/min/1.73 m^2^)	8.7 ± 3.4	10.4 ± 4.6	0.003
PTH (pg/mL)	233.2 ± 100.9	277.2 ± 104.9	0.135
Ferritin (ng/mL)	603.0 ± 221.0	333.7 ± 185.7	0.009
CRP (mg/dL)	7.3 ± 2.4	2.5 ± 1.7	<0.001
Sepsis at dialysis start—*n* (%)	62 (57.9)	24 (22.4)	<0.001
Vascular access at dialysis start—*n* (%)			
Central venous catheter	82 (76.6)	62 (66.0)	0.18
Arteriovenous fistula	26 (24.3)	30 (31.9)	0.187
Arteriovenous graft	2 (1.9)	2 (2.1)	0.874
Outcomes			
One-year mortality—*n* (%)	38 (35.5)	18 (20.5)	0.021

**Table 3:** Univariate and multivariate analyses of factors predictive of early mortality.

Characteristic	Mortality			
	Unadjusted OR (95% CI)	*p*-Value	Adjusted OR (95% CI)	*p*-Value
Age	1.08 (1.04–1.13)	<0.001	1.07 (1.03–1.12)	0.002
Gender (Male)	0.87 (0.47–1.63)	0.672		
Race (Caucasian)	2.55 (0.55–11.78)	0.230		
Comorbidities				
Hypertension	0.54 (0.22–1.34)	0.185		
Diabetes	0.76 (0.41–1.43)	0.401		
Ischemic cardiomyopathy	1.21 (0.65–2.25)	0.554		
COPD	1.31 (0.60–2.84)	0.499		
Cerebrovascular disease	1.08 (0.51–2.31)	0.841		
Peripheral artery disease	1.13 (0.56–2.29)	0.735		
Dementia	0.93 (0.24–3.63)	0.913		
Neoplasia	1.37 (0.61–3.08)	0.440		
Chronic liver disease	0.82 (0.16–4.20)	0.813		
Charlson Index	1.22 (1.04–1.44)	0.017	1.10 (0.91–1.35)	0.324
Laboratory at dialysis start				
Haemoglobin	0.99 (0.81–1.21)	0.923		
Serum Urea	1.00 (0.99–1.01)	0.769		
Serum Creatinine	0.80 (0.65–0.97)	0.022	0.80 (0.64–0.99)	0.049
eGFR	1.06 (0.98–1.14)	0.139		
Albumin	0.38 (0.22–0.66)	0.001	0.41 (0.22–0.75)	0.004
PTH	1.00 (0.99–1.00)	0.735		
Ferritin	1.00 (1.00–1.00)	0.208		
CRP	1.04 (0.99–1.09)	0.075		
Central venous catheter at dialysis start	2.09 (0.99–4.41)	0.053		
Sepsis at dialysis start	1.87 (1.00–3.57)	0.050		

## Data Availability

The data presented in this study are available on request from the corresponding author.
